# Supplementary material for: The Dynamics of Lepus granatensis and Oryctolagus cuniculus in a Mediterranean Agrarian Area: Are Hares Segregating from Rabbit Habitats after Disease Impact?
Source: Animals (Basel). 2022 May 25;12(11):1351. doi: 10.3390/ani12111351 (PMC9179464; doi:10.3390/ani12111351)
Supplement: Supplementary file 1 [file animals-12-01351-s001.zip › animals-1724415-supplementary.pdf]

**Table S1.** Correlations between the frequency distributions of hourly records with the available for 1990-2019 period.

| | Chi-Square | df | p |
|---|---|---|---|
| **hare / available** | 24.03574 | 23 | p < 0.401838 |
| **rabbit / available** | 17.24271 | 23 | p < 0.797169 |
| **hare / rabbit** | 77.13317 | 23 | p < 0.000000 |

**Table S2.** Correlations between the frequency distributions of hourly records with the available for 2016-2020 period (quantitative results only).

| | Chi-Square | df | p |
|---|---|---|---|
| **hare / available** | 27.41574 | 23 | p < 0.238696 |
| **rabbit / available** | 17.63803 | 23 | p < 0.776812 |
| **hare / rabbit** | 55.87575 | 23 | p < 0.000147 |

**Table S3.** Spatial correlations for 1995-2019 period (PCA distribution).

| | FECHA | LgUTMx | LgUTMy | LgALT | FACTOR 1 | FACTOR 2 | FACTOR 3 |
|---|---|---|---|---|---|---|---|
| **LgUTMx** | **-0.0939**<br>**N=535**<br>**p=0.030** | | | | | | |
| **LgUTMy** | **0.41**<br>**N=535**<br>**p=0.00** | **-0.2**<br>**N=535**<br>**p=0.000** | | | | | |
| **LgALT** | **0.1859**<br>**N=535**<br>**p=0.000** | 0.0212<br>N=535<br>p=0.624 | **0.4553**<br>**N=535**<br>**p=0.00** | | | | |
| **FACTOR 1** | **-0.3593**<br>**N=535**<br>**p=0.000** | **0.3732**<br>**N=535**<br>**p=0.00** | **-0.874**<br>**N=535**<br>**p=0.00** | **-0.7748**<br>**N=535**<br>**p=0.00** | | | |
| **FACTOR 2** | -0.0087<br>N=535<br>p=0.840 | **0.8974**<br>**N=535**<br>**p=0.00** | -0.0244<br>N=535<br>p=0.573 | **0.4597**<br>**N=535**<br>**p=0.00** | 0<br>N=535<br>p=1.00 | | |
| **FACTOR 3** | **-0.2038**<br>**N=535**<br>**p=0.000** | **-0.2356**<br>**N=535**<br>**p=0.000** | **-0.4853**<br>**N=535**<br>**p=0.00** | **0.434**<br>**N=535**<br>**p=0.00** | 0<br>N=535<br>p=1.00 | 0<br>N=535<br>p=1.00 | |
| **Lg Lg** | **-0.4676**<br>**N=152**<br>**p=0.000** | **0.1862**<br>**N=152**<br>**p=0.022** | **-0.3635**<br>**N=152**<br>**p=0.000** | -0.0211<br>N=152<br>p=0.797 | **0.3003**<br>**N=152**<br>**p=0.000** | **0.1673**<br>**N=152**<br>**p=0.039** | **0.287**<br>**N=152**<br>**p=0.000** |
| **Lg Oc** | **-0.2147**<br>**N=186**<br>**p=0.003** | 0.1054<br>N=186<br>p=0.152 | -0.0256<br>N=186<br>p=0.729 | 0.0457<br>N=186<br>p=0.535 | 0.0189<br>N=186<br>p=0.798 | 0.1154<br>N=186<br>p=0.117 | 0.0162<br>N=186<br>p=0.826 |

**Table S4.** Variance coefficient explained by each period in the PCA of the spatial analysis for 1995-2019 period.

| | Mean | | CV (%) | |
|---|---|---|---|---|
| | hare | rabbit | hare | rabbit |
| **Factor 1** | **0.362** | **-0.232** | **267.6** | **566.3** |
| **Factor 2** | **-0.226** | **0.145** | **305.9** | **790.0** |
| **Factor 3** | **-0.318** | **0.204** | **183.6** | **333.0** |
| **AMPLITUDE** | | | **801.5** | **1814.7** |

**Table S5.** T-test with species as grouping variable for the PCA.

| | **Mean** *Lepus granatensis* | **Mean** *Oryctolagus cuniculus* | **t-value** | **df** | **p** | **Valid N** *Lepus granatensis* | **Valid N** *Oryctolagus cuniculus* |
|---|---|---|---|---|---|---|---|
| **DATE** | 01/06/2014 | 28/06/2015 | -2.5121 | 533 | **0.012297** | 209 | 326 |
| **LgUTMx** | 5.41 | 5.41 | 0.3222 | 533 | 0.74739 | 209 | 326 |
| **LgUTMy** | 6.62 | 6.62 | -0.5478 | 533 | 0.584065 | 209 | 326 |
| **LgALT** | 1.8 | 1.94 | **-10.8712** | 533 | **0** | 209 | 326 |
| **FACTOR 1** | 0.36 | -0.23 | 5.627 | 533 | **0** | 209 | 326 |
| **FACTOR 2** | -0.23 | 0.14 | -4.2164 | 533 | **0.000029** | 209 | 326 |
| **FACTOR 3** | -0.32 | 0.2 | -9.1529 | 533 | **0** | 209 | 326 |

**Table S6.** Spatial correlations for 2016-2020 period (PCA distribution).

| | **FECHA** | **LgUTMx** | **LgUTMy** | **LgALT** | **LgLg** | **LgOc** | **FACTOR 1** | **FACTOR 2** |
|---|---|---|---|---|---|---|---|---|
| **LgUTMx** | **0.2375** N=5867 p=0.00 | | | | | | | |
| **LgUTMy** | **-0.1022** N=5867 p=0.000 | **-0.4378** N=5867 p=0.00 | | | | | | |
| **LgALT** | **-0.0267** N=5867 p=0.041 | **-0.5611** N=5867 p=0.00 | **0.8936** N=5867 p=0.00 | | | | | |
| **LgLg** | -0.0311 N=88 p=0.774 | 0.0731 N=88 p=0.498 | 0.0702 N=88 p=0.516 | 0.011 N=88 p=0.919 | | | | |
| **LgOc** | **-0.2962** N=132 p=0.001 | -0.1334 N=132 p=0.127 | -0.1519 N=132 p=0.082 | -0.1466 N=132 p=0.093 | -- N=0 p= --- | | | |
| **FACTOR 1** | **0.2228** N=5866 p=0.00 | **0.7439** N=5866 p=0.00 | **-0.9053** N=5866 p=0.00 | **-0.9394** N=5866 p=0.00 | 0.0013 N=88 p=0.991 | 0.0362 N=132 p=0.680 | | |
| **FACTOR 2** | **-0.94** N=5865 p=0.00 | **-0.2442** N=5865 p=0.00 | **-0.1902** N=5865 p=0.00 | **-0.2343** N=5865 p=0.00 | -0.0045 N=88 p=0.967 | **0.3425** N=132 p=0.000 | 0.0013 N=5864 p=0.919 | |
| **FACTOR 3** | **0.2554** N=5865 p=0.00 | **-0.6187** N=5865 p=0.00 | **-0.3242** N=5865 p=0.00 | **-0.1166** N=5865 p=0.00 | -0.1081 N=88 p=0.316 | 0.1021 N=132 p=0.244 | -0.0003 N=5864 p=0.980 | 0.0001 N=5863 p=0.994 |

**Table S7.** Eigenvalues of correlation matrix. and related statistics for 2016-2020 period (PCA distribution).

| Factor | Eigenvalue | % Total variance | Cumulative Eigenvalue | Cumulative % |
|---|---|---|---|---|
| 1 | 2.339 | 57.8 | 2.3 | 57.8 |
| 2 | 1.03 | 25.9 | 3.3 | 83.7 |
| 3 | 0.57 | 14.2 | 3.9 | 97.8 |
| 4 | 0.09 | 2.2 | 4 | 100 |

**Table S8.** Factor coordinates of the variables. based on correlations for 2016-2020 period (PCA distribution).

| | Factor 1 | Factor 2 | Factor 3 | Factor 4 |
|---|---|---|---|---|
| **DATE** | 0.22 | **-0.94** | 0.26 | 0.03 |
| **LgUTMx** | **0.75** | -0.24 | **-0.62** | -0.05 |
| **LgUTMy** | **-0.91** | -0.19 | -0.32 | 0.19 |
| **LgALT** | **-0.94** | -0.23 | -0.12 | -0.22 |

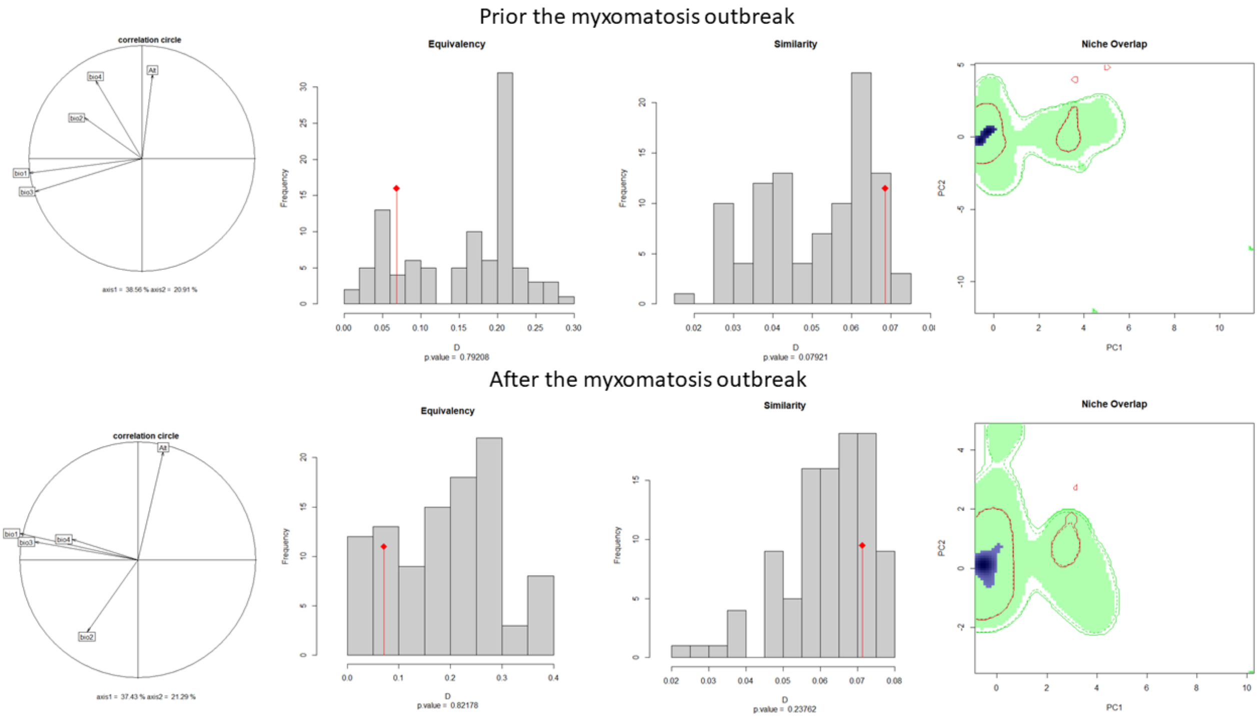


**Figure S1.** Niche PCA. overlap. equivalency and similarity analyses prior and after the disease outbreak in rabbit (*Oryctolagus cunniculus*) and Iberian hare (*Lepus granatensis*). In the PCA plot red line indicates the niche used by the 75% of records.
